# SARS-CoV-2 replicon for high-throughput antiviral screening

**DOI:** 10.1099/jgv.0.001583

**Published:** 2021-05-06

**Authors:** Qiu-Yan Zhang, Cheng-Lin Deng, Jing Liu, Jia-Qi Li, Hong-Qing Zhang, Na Li, Ya-Nan Zhang, Xiao-Dan Li, Bo Zhang, Yi Xu, Han-Qing Ye

**Affiliations:** ^1^​ The Joint Center of Translational Precision Medicine, Guangzhou Institute of Pediatrics, Guangzhou Women and Children’s Medical Center, Guangzhou, 510623, PR China; ^2^​ The Joint Center of Translational Precision Medicine, Wuhan Institute of Virology, Chinese Academy of Sciences, Wuhan, 430071, PR China; ^3^​ Key Laboratory of Special Pathogens and Biosafety, Wuhan Institute of Virology, Center for Biosafety Mega-Science, Chinese Academy of Sciences, Wuhan, PR China; ^4^​ University of Chinese Academy of Sciences, Beijing 100049, PR China; ^5^​ Hunan Normal University, School of Medicine, Changsha, 410081, PR China

**Keywords:** SARS-CoV-2, replicon, antiviral screening

## Abstract

The severe acute respiratory syndrome coronavirus-2 (SARS-CoV-2) virus, which is highly pathogenic and classified as a biosafety level 3 (BSL-3) agent, has greatly threatened global health and efficacious antivirals are urgently needed. The high requirement of facilities to manipulate the live virus has limited the development of antiviral study. Here, we constructed a reporter replicon of SARS-CoV-2, which can be handled in a BSL-2 laboratory. The Renilla luciferase activity effectively reflected the transcription and replication levels of the replicon genome. We identified the suitability of the replicon in antiviral screening using the known inhibitors, and thus established the replicon-based high-throughput screening (HTS) assay for SARS-CoV-2. The application of the HTS assay was further validated using a few hit natural compounds, which were screened out in a SARS-CoV-2 induced cytopathic-effect-based HTS assay in our previous study. This replicon-based HTS assay will be a safe platform for SARS-CoV-2 antiviral screening in a BSL-2 laboratory without the live virus.

The newly emerging severe acute respiratory syndrome coronavirus-2 (SARS-CoV-2), which caused COVID-19 respiratory disease, has infected more than 120 million people including over 2.6 million mortalities worldwide (https://www.worldometers.info/coronavirus/). Currently, the infection cases are increasing rapidly, but no efficient antiviral drug and efficacious therapeutics are available for SARS-CoV-2 infection. The high-throughput screening (HTS) platform, which allows rapid antiviral screening from large-scale compound libraries, would facilitate the discovery of new antivirals. Recently, the HTS assays based on virus-induced cytopathic effects (CPEs) and reporter virus have been developed for antiviral study for SARS-CoV-2 [1, 2]. As SARS-CoV-2 is classified as biosafety level 3 (BSL-3) agents, the high demand of facilities to manipulate the live virus limits the application of the above HTS assays. Therefore, the construction of a noninfectious replicon would provide a reliable and safe tool to establish a HTS assay that can be performed in BSL-2 laboratories.

The genome of SARS-CoV-2 contains a single positive-sense RNA, which is approximately 29.9 kb in length. The first two-thirds of the genome encode the nonstructural proteins, which generate a replicase-transcriptase complex that participates in genome RNA replication and subgenomic mRNA transcription. The remaining one-third of the genome encodes four structural proteins S, E, M and N, and five accessory proteins NS3, NS6, NS7A, NS7B and NS8. The structural proteins form the virus particles, and have significant roles in virus fusion, entry, assembly and budding. The replicons contain the intact nonstructural protein genes but lack the structural protein genes, and therefore are self-replicable but cannot produce infectious viral particles, making them a safe tool for demonstrating viral replication processes and antiviral agents' screening. The goal of this study was to develop the replicon-based HTS assay for SARS-CoV-2 to facilitate the antiviral screening in a BSL-2 laboratory.

Our strategy for the construction of SARS-CoV-2 replicon was similar to that of SARS-CoV replicon [3]. The nucleotides 21593–28213 of SARS-CoV-2 (WIV04, GenBank No: MN996528.1) [4] genome, which include the coding sequences of S, E, M and all the accessory proteins, were deleted, and the *Renilla* luciferase (Rluc) reporter gene was engineered downstream the transcription regulatory sequence (TRS) of the S gene (Fig. 1a). The ORF encoding the nucleocapsid N was retained as it was reported to enhance viral RNA replication. The T7 promoter and a poly(A)_21_ tail were introduced upstream the 5′ terminus and downstream the 3′ terminus of the replicon, respectively. Eleven contiguous cDNA fragments covering the entire replicon were chemically synthesized and cloned into the pACYC177 vector to generate the intermediate subclones. The intermediate fragments were assembled step-by-step into a modified pBeloBAC vector by using the unique restriction sites (Fig. S1, available in the online version of this article). The obtained replicon clone (SARS-CoV-2 replicon-WT) was linearized and used as a template for *in vitro* transcription to produce the replicon RNA.

**Fig. 1.**
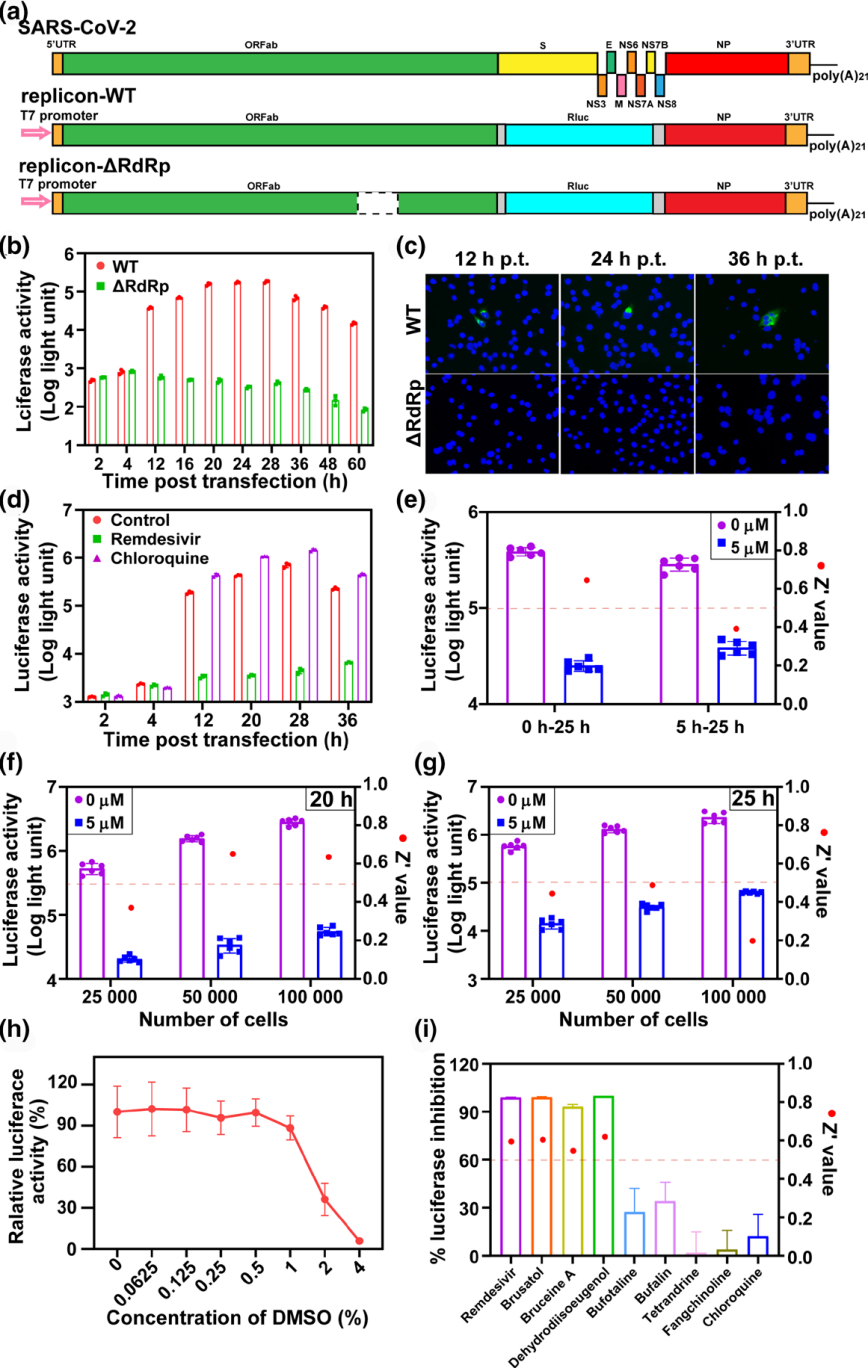
Establishment, optimization and application of the SARS-CoV-2 replicon-based HTS assay. (a) Strategy schematic of the wild-type (WT) and ΔRdRp mutant SARS-CoV-2 replicons. In the WT replicon, the S, E, M and the accessory protein genes within the SARS-CoV-2 genome were deleted. The transcription regulatory sequences (TRS) of the S protein were retained, from which the Rluc reporter gene was under control, and the 60 nt upstream the ORF of N was retained. ΔRdRp mutant replicon contains a 339–934 aa deletion within the nsP12 gene. (b) Luciferase activities of the cells electroporated with the WT and ΔRdRp mutant replicon RNAs at the indicated time points. The results of three independent experiments are presented. (c) Detection of the expression of NP protein in the WT or ΔRdRp mutant replicons transfected cells at different time post-electroporation by IFA. (d) The effects of remdesivir and chloroquine on viral RNA replication. The BHK-21 cells were electroporated with 15 µg WT replicon, and immediately treated by 10 µM remdesivir and chloroquine, and 0.05 % DMSO as control, respectively. The results of three independent experiments are presented. (e) Comparison of time points of adding compounds. The WT replicon electroporated cells were seeded along with 5 µM remdesivir treatment (0 h) or seeded 5 h prior to compound treatment (5 h). The Rluc signals were detected after 25 h treatment. (f–g) Optimization of the cell number and incubation time for the HTS assay. The WT replicon electropated cells were seeded at different densities of 25 000, 50 000 and 100 000 per well along with treatment of remdesivir (5 µM) or DMSO (0.05 %), and the Rluc signals were read at 20 h post-transfection (p.t.) (f) or 25 h p.t. (g), respectively. (h) Effects of DMSO on SARS-CoV-2 replicon replication. The replicon electroporated cells were treated with different concentrations of DMSO. Luciferase activities were determined at 20 h post-treatment. (i) Screening of several natural compounds using the replicon-based HTS assay. Fourteen natural compounds among the 30 hits showing antiviral activities in our previous findings were subjected to the replicon-based HTS assay. The results of seven representative compounds were presented. The replicon transfected cells were treated with individual compound at the concentration exhibiting >90 % inhibitory effect on virus infection. The Rluc signals were examined at 20 h p.t. The Z’ values were calculated against DMSO treatment.

To analyse the replication ability of the replicon, we constructed a mutant replicon-ΔRdRp with 339–934 aa deletion within the RNA-dependent RNA polymerase nsP12 as a negative control (Fig. 1a). After electroporation into BHK-21 cells, the transcription and replication of the replicon RNAs were evaluated by the expression of Rluc and N proteins. As shown in Fig. 1b, c, in the WT replicon transfected cells, the Rluc activity continued to increase from 4 to 28 h p.t., and sporadic N-positive cells were observed from 12 to 36 h p.t., indicating the transcription and replication of WT replicon without producing infectious viruses. For the replicon-ΔRdRp, only the background level of Rluc signal was detected and no IFA positive cell was generated, suggesting that the deletion of RdRp abolished genome replication.

We next validated the application of replicon for drug discovery using two known inhibitors of SARS-CoV-2, remdesivir and chloroquine [5]. The replicon transfected cells were treated with 10 µM remdesivir and chloroquine, respectively. The Rluc activities were significantly suppressed by remdesivir but not chloroquine from 12 to 36 h p.t. (Fig. 1d). This result was consistent with previous study that remdesivir inhibits viral RNA synthesis as an adenosine analogue, and chloroquine blocks virus infection primarily by suppressing virus entry [5]. We also compared the antiviral ability of remdesivir on replicon and infectious virus. The EC_50_ values calculated by Rluc signal reduction of the replicon and viral RNA copies reduction of the virus were 0.36 µM and 0.92 µM, respectively (Fig. S2), which confirmed the sensitivity of replicon for antiviral screening. Collectively, these results demonstrated that the SARS-CoV-2 replicon is a reliable and safe tool for screening antiviral drugs targeting viral RNA transcription and replication.

To develop the SARS-CoV-2 replicon into a HTS assay, we identified the appropriate parameters including cell density, and drug incubation time in 96-well plate format using remdesivir as a positive control and DMSO as a negative control. The Z’ values were calculated to evaluate the assay quality (Z’ value ≥0.5 is considered robust for HTS assay). As shown in Fig. 1e, remdesivir markedly inhibited the Rluc activities when the replicon transfected cells were seeded either along with the drug at the same time or 5 h prior to drug treatment. However, plating cells and compound simultaneously yielded a Z’>0.5 value, so this method was chosen for the following tests. Next, different numbers of the replicon transfected cells (25 000, 50 000 or 100 000) were seeded into each well with or without treatment of remdesivir, and the Rluc signals were read at 20 h or 25 h p.t. By comparing the Rluc signals and Z’ values among above different conditions (Fig. 1g, f), we chose the cell density at 50 000 and the detection time point of Rluc signal at 20 h p.t. as the optimized condition for the replicon-based HTS assay in a 96-well plate. Under this condition, the Z’ value of remdesivir was 0.65, implying the robust quality of HTS assay.

As the compounds are usually dissolved in DMSO, to exclude the effect of DMSO on the replicon, the replicon transfected cells were treated with different concentrations of DMSO and detected the Rluc signals at 20 h p.t. under above condition. Fig. 1h showed that DMSO had no influence on the Rluc activities at ≤1 % (v/v) concentration. Therefore, we determined 0.05 % DMSO as the optimal concentration to dissolve the compounds in HTS assay.

We had recently screened out 30 natural compounds showing antiviral activity against SARS-CoV-2 through a virus-induced CPE-based HTS assay [1]. Here, 14 of them were selected to further test and validate the availability of replicon and demonstrate which of these compounds function at the replication stage (Table S1). For each compound, the working concentration at which the inhibitor exhibited >90 % inhibitory effect on SARS-CoV-2 infection without cytotoxity was used. Three compounds, brusatol, bruceine A, and dehydrodiisoeugenol displayed >90 % inhibitory effects on the Rluc activities with Z’ values of 0.6, 0.55 and 0.62, respectively (Fig. 1i), suggesting that these compounds are viral RNA synthesis inhibitors for SARS-CoV-2. Seven drugs (bufotaline, cinobufagin, bufalin, digoxin, cornuside, roburicacid and dehydrocostus lactone) had moderate inhibitory effects ranging from 27.3–61.4 %, indicating that these drugs partially suppress viral RNA replication and may affect other stages of viral life cycle. Four of them (tetrandrine, fangchinoline, isoliensinine and veratridine) showed <10 % reduction in Rluc signals (Table S1), suggesting that they do not exert directly on viral RNA replication.

Among the three RNA synthesis inhibitors, bruceine A is a potential candidate for the treatment of canine babesiosis, brusatol is an Nrf2 inhibitor, and dehydrodiisoeugenol has anti-inflammatory and anti-bacterial activities. Our results provide the first evidence and mechanism for the antiviral effects of bruceine A and dehydrodiisoeugenol. Brusatol has been reported to suppress HCV infection at the RNA level. Here, we also identified that brusatol prevents SARS-CoV-2 infection at the stage of viral RNA replication. Tetrandrine and fangchinoline are bisbenzylisoquinoline alkaloids and calcium channel blockers. Both of the two compounds could inhibit the human coronavirus OC43 at the early stage of infection and the MERS-CoV pseudovirus translocation [6]. Our results demonstrated that they could effectively suppress the SARS-CoV-2 infection without affecting viral RNA synthesis (Fig. 1i, Table S1). These findings further confirmed that tetrandrine and fangchinoline are the viral entry inhibitors for coronaviruses. Therefore, the replicon assay could facilitate to analyse the antiviral mechanism of the compounds.

In summary, we had constructed a SARS-CoV-2 replicon, which could represent the viral RNA transcription and replication process and screen antiviral compounds. The replicon-based HTS assay was developed, which could be used for rapid and large-scale antiviral screening in a BSL-2 laboratory.

## Data availability

The data used and analysed in this study are available in the main text and the Supplementary Material. Any other raw data that support the findings of this study are available from the corresponding author upon reasonable request.

## Supplementary Data

Supplementary material 1
