# Supplementary material for: SARS-CoV-2 replicon for high-throughput antiviral screening
Source: J Gen Virol. 2021 May 6;102(5):001583. doi: 10.1099/jgv.0.001583 (PMC8295919; doi:10.1099/jgv.0.001583)
Supplement: Supplementary material 1 [file jgv-102-1583-s001.pdf]

Supplementary Materials for

SARS-CoV-2 replicon for high-throughput antiviral screening

Qiu-Yan Zhang[1,2,†], Cheng-Lin Deng[3,†], Jing Liu[3,4†], Jia-Qi Li[3,4†], Hong-Qing Zhang[3,4†], Na Li[3,4†], Ya-Nan Zhang[3,4†], Xiao-Dan Li[5,*], Bo Zhang[1,2,3,*], Yi Xu[1,2,*], Han-Qing Ye[3,*]

Correspondence to: *lxd@live.cn* or *zhangbo@wh.iov.cn* or *xuyi70@163.com* or *yehq@wh.iov.cn*

This PDF file includes:

Materials and Methods

Figures. S1 to S2

Tables S1

References

Materials and Methods

Cell lines, antibodies and reagents

BHK-21 cells (ATCC number CCL-10) and Vero-E6 cells (ATCC number CRL-1686) were cultured at 37 °C with 5% CO_2 in Dulbecco's modified Eagle's medium (DMEM; Gibco, Thermo fisher scientific, America) complemented with 10 % FBS, 100 U/ml penicillin and 100 mg/ml streptomycin. The rabbit anti-RP3-CoV NP protein antibody which is cross-reactive with the NP protein of SARS-CoV-2 was kindly provided by Prof. Zheng-Li, Shi (Wuhan institute of Virology, CAS). FITC-conjugated goat anti-rabbit IgG were purchased from Protein Tech Group. Remdsivir and chloroquine were purchased from Selleck, and other natural compounds were purchased from Weikeqi Biotech (Sichuan, China).

Plasmid construction

To construct a reporter-containing replicon of the SARS-CoV-2 (WIV04, Genbank No. MN996528.1), the nucleotides 21593-28213 of the genome containing the S, E, M and all the accessory proteins, except N were deleted and replaced by the *renilla* luciferase (Rluc) reporter gene, making the Rluc gene under control of the transcription regulation sequence (TRS) of S gene. Eleven fragments ranging from 1.5-2.8 kb covering the entire replicon genome were chemically synthesized and cloned into pUC57 vector by Sangon biotech. To maintain the gene stability in the bacteria, all of these fragments were cloned into low-copy number vector pACYC-177, and assembled step-by-step using the specific restriction sites in the genome into four larger subclones, which are pACYC-F1, pACYC-F2, pACYC-F3 and pACYC-F4, respectively. A T7 promoter and a poly(A)$_{21}$ tail were added upstream F1 and downstream F4 fragments, respectively. The bacterial artificial chromosome (BAC) vector was used to carry the entire replicon. To facilitate the assembly, pBeloBAC11 vector was firstly modified to insert *Aat*II restriction enzyme site and to retain a single *Not*I restriction enzyme site. The subclone pACYC-F1 and F2, pACYC-F3 and F4 were firstly assembled into modified pBeloBAC11 to obtain

pBeloBAC-F1+F2 and pBeloBAC-F3+F4, respectively. Then the F1+F2 fragment was ligated into F3+F4 with *Aat*II/*Mlu*I enzyme sites to obtain the wild-type full length clone of replicon (SARS-CoV-2rep-WT). The ΔRdRp deletion fragment which contains the 339-934 aa deletion within nsP12 protein was amplified by overlapping PCR and introduced into the wild type SARS-CoV-2rep-WT with *Mlu*I and *BstB*I restriction sites. All constructs were verified by DNA sequencing.

Transcription and electroporation

The wild type and ΔRdRp mutant replicon clones were linearized with *Not*I and subjected to *in vitro* transcription using T7 mMESSAGE mMACHINE kit (Invitrogen) according to the manufacturer's instruction with some modifications. For 5 h at 37 °C, 50 μL reaction was performed by adding 5 μg linearized DNA and 7.5 μL GTP stock, resulting in a 1:1 ratio of GTP to cap analog. After removing the template DNA by nuclease, the obtained RNAs were precipitated by Lithium chloride (LiCl) and dissolved in Nuclease-free water. Approximately 15 μg RNA transcripts were electroporated into 8×10^6 BHK-21 cells suspended in 0.8 mL ice-cold PBS in a 0.4 cm cuvette with a GenePulser apparatus (Bio-Rad) at 850 V and 25 μF, pulsing three times at 3s intervals. After 10 min recovery at room temperature, the electroporated cells were mixed with 20 mL pre-warmed DMEM containing 10 % FBS and then seeded in 6-well, 12-well or 96-well plates with different cell densities.

Luciferase assay

The eletroporated cells seeded in the 12-well or 96-well plates were lysed with 1×Rluc lysis buffer (Promega) at the indicated time points. Rluc activity was measured using a Multimode Microplate Reader (Varioskan Flash, Thermo Fisher, Finland) by mixing 20 μL cell lysates with 50 μL substrate (Promega).

Indirect immunofluorescence assay

The electroporated cells seeded in 6-well plate were fixed at different time points using cold acetone in methanol (5%) for 10 minutes and washed with PBS three times. Then, the fixed cells were incubated with anti-NP protein (1:1000 dilutions in PBS) for 1 h. After washing with PBS three times, the cells were incubated with goat anti-rabbit IgG conjugated to FITC at room temperature for another hour. After three

times PBS washes, the slides were mounted with 95 % glycerol and examined under a fluorescence microscope. Cell images were taken at ×400 magnification.

Antiviral assay

For the replicon based assay, when electroporated cells were seeded in 12-well plates, remdesivir was added at different concentrations. After 28 h, the cells were lysed and Rluc activity was detected. For the virus based assay, Vero-E6 cells were seeded in 24-well plates (8×10^4 cells per well). The cells were infected with SARS-CoV-2 (MOI=0.01) and incubated with different concentrations of remdesivir at 37 °C for 24 h. Subsequently, the viral RNAs were extracted from the cell culture medium from each well and subjected to qRT-PCR assay. qRT-PCR was performed as indicated before (1). The antiviral activity of remdesivir was expressed as 50% effective concentration (EC_{50}) which was calculated by GraphPad Prism software 8.0.

HTS assay

HTS assay was developed in 96-well plate format using remdesivir as positive control and 0.05% DMSO as negative control. All compounds were dissolved in dimethyl sulfoxide (DMSO) followed by diluted with DMEM medium containing 2% FBS. 5×10^4 electroporated cells (total volume 250 μL) were seeded per well along with different concentrations of compounds. After 20 h incubation, the cells were washed with PBS three times and lysed to detect the Rluc signals.

Statistical analysis

Statistical analysis of Z' -values were calculated as follows: $Z' = 1 - (3SD \text{ of sample} + 3SD \text{ of control})/|\text{Mean of sample} - \text{Mean of control}|)$. Here, SD is the standard deviation of the luciferase signals from cell control or sample. Z′ values between 0.5 and 1 are considered good quality.

Figure S1.

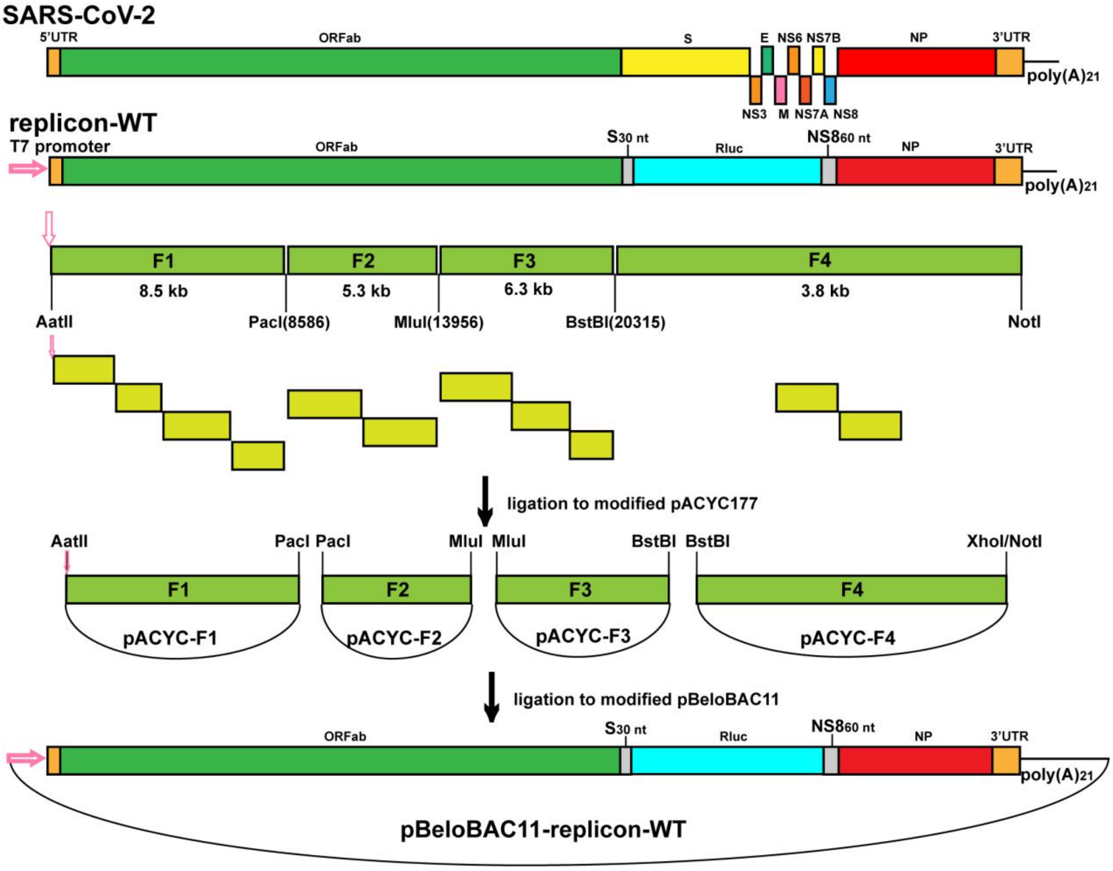

Figure S1. Scheme of the strategy to construct the SARS-CoV-2 replicon. Eleven fragments covering the full length of replicon were chemically synthesized and cloned into pACYC-177 plasmid. The individual subclone was assembled into four larger fragments F1, F2, F3 and F4. A T7 promoter and a poly(A)$_{21}$ tail were engineered at the 5' terminal of F1 and 3' terminal of F4 fragment, respectively. The F1, F2, F3 and F4 fragments were assembled step-by-step into a modified pBeloBAC11 vector to obtain the SARS-CoV-2 replicon-WT clone.

Figure S2.

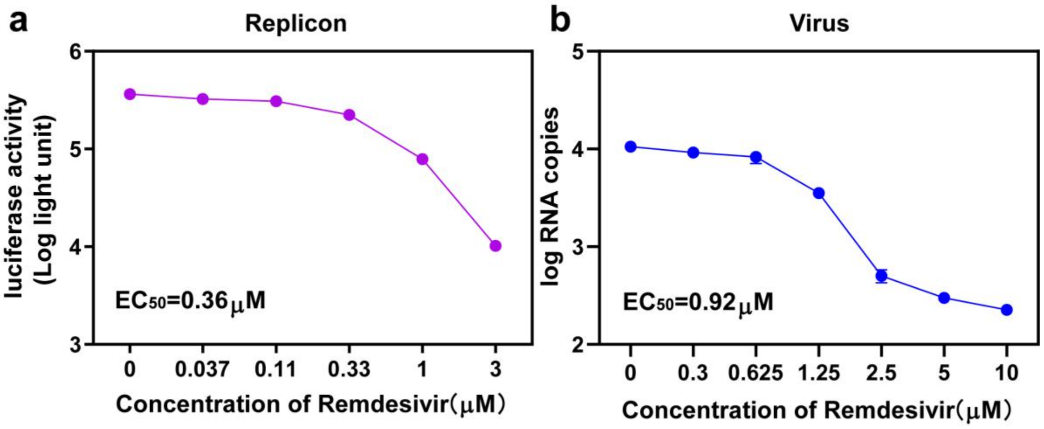

Figure S2. Remdesivir inhibited replication of SARS-CoV-2 virus and replicon system. (a) The SARS-CoV-2 replicon electroporated BHK-21 cells were mixed with different concentrations of remdesivir, and plated into 12-well plates. At 28 h post electroporation, the cells were lysed and the Rluc signals were detected. (b) Vero-E6 cells were infected with SARS-CoV-2 virus at an MOI of 0.01 with different concentrations of remdesivir, and at 24 h post infection, the viral RNAs were extracted from the supernatants and subjected to qRT-PCR for verification of virus RNA copies. 50% effective concentration (EC_{50}) was calculated by GraphPad Prism software 8.0.

Table S1

Hit compounds	Resources	Structure	Concentration of testing (μM)	% inhibition in replicon	% inhibition in virus
Bufotaline	Toad Venom	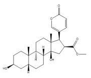	0.5	27.3	>90
Cinobufagin	Toad Venom	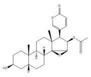	0.5	34.1	>90
Brusatol	Brucea javanica	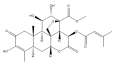	0.5	98.9	>90
Bruceine A	Brucea javanica	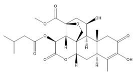	0.5	93.2	>90
Bufalin	Toad Venom	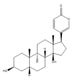	0.5	34.0	>90
Digoxin	Digitalis	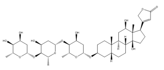	5	39.5	>90
Tetrandrine	Stephania tetrandrine	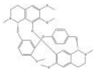	5	1.8	>90
Fangchinoline	Stephania tetrandrine	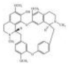	5	3.8	>90
Isoliensinine	Nelumbo nucifera	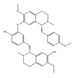	10	1.0	>90
Veratridine	Lily family	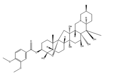	10	6.3	>90
Cornuside	Cornus officinalis	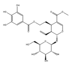	10	46.4	>90
Roburicacid	Gentiana macrophylla	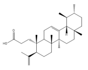	10	54.5	>90
Dehydrocostus lactone	Saussurealappa	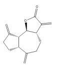	10	61.4	>90
Dehydrodiisoeugenol	Aristolochia taliscana	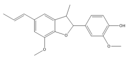	40	99.8	>90

Table S1. The table shows the resources, structures and concentration of the tested compounds and the inhibition rate on SARS-CoV-2 replicon and viruses.

References

1. **Zhang ZR, Zhang YN, Li XD, Zhang HQ, Xiao SQ, Deng F, Yuan ZM, Ye HQ, Zhang B.** 2020. A cell-based large-scale screening of natural compounds for inhibitors of SARS-CoV-2. Signal transduction and targeted therapy **5:**218.
